# Development of the TIFFIN recommendations for co-producing palliative and end-of-life care research with individuals with lived experience of homelessness: A qualitative study

**DOI:** 10.1177/02692163241259667

**Published:** 2024-06-19

**Authors:** Jodie Crooks, Kate Flemming, Caroline Shulman, Emma Casey, Briony Hudson

**Affiliations:** 1 Marie Curie, London, UK; 2 University of York, York, UK; 3 Pathway, London, UK; 4 Marie Curie Palliative Care Research Department, University College London, London, UK; 5 Groundswell, London, UK

**Keywords:** Palliative care, homelessness, qualitative research, patient participation, community participation

## Abstract

**Background:**

Palliative care for people experiencing homelessness is a complex field. Due to the intricate nuances and heterogeneity in the experience of palliative care for people without secure housing, it is essential that research is informed by people with lived experience of homelessness. However, as homelessness is often associated with loss, trauma and high levels of exposure to death, any co-production of research, particularly in the field of palliative and end-of-life-care, must be trauma-informed.

**Aim:**

To produce recommendations for co-producing palliative and end-of-life-care research with people with lived experience of homelessness.

**Design:**

A qualitative study comprising semi-structured interviews and focus groups. Data were analysed using iterative, reflexive thematic analysis.

**Setting/participants:**

Twenty-seven participants were recruited. Sixteen professionals with experience of co-producing research with people with lived experience of homelessness; eleven people with lived experience of homelessness.

**Results:**

Six key themes were developed: transparency, importance of engagement and rapport, facilitating equitable involvement via person centred approach, financial recognition of involvement, involvement and growth through a trauma-informed approach and navigating institutional resistance and attitudes. Recommendations corresponding to the core themes were developed (TIFFIN recommendations).

**Conclusions:**

Co-production of palliative care research with people with lived experience of homelessness is essential, but must be done carefully and sensitively. As a population with high levels of premature morbidity and mortality yet low access to palliative care, the TIFFIN recommendations could help to support the involvement of people with lived experience of homelessness in palliative and end-of-life-care care research.

**What is already known about the topic?**

- Research into palliative care for people experiencing homelessness is complex and requires input from people with lived experience.
- There is a dearth of evidence and/or guidance in how to support researchers to involve people with lived experience of homelessness in palliative care research.

**What this paper adds?**

- Co-production of palliative and end-of-life-care research with people with lived experience of homelessness needs to be transparent, prioritise building rapport, be trauma-informed and person-centred.
- Reimbursement should always be offered to co-producers. The method of reimbursement should consider the context of involvement (i.e. the individual’s circumstances) where possible.
- There is a need to evidence the impact of involvement, to facilitate a change in research culture which prioritises hearing the voices of different groups.

**Implications for practice, theory or policy**

- Involving people with lived experience of homelessness can help researchers to identify unknown unknowns within the field of palliative care: it can validate, enhance and direct research to the intricacies of their experiences.
- The TIFFIN recommendations provide guidance for how to achieve co-production within this field in a trauma informed way.

## Background

People experiencing homelessness experience premature mortality with age-adjusted death rates around four times higher than the housed population.^1^ Many barriers to healthcare access are experienced by this group, which can be linked to previous traumatic experiences, mental health and/or substance misuse issues. Many people experiencing homelessness die unsupported in undignified situations.^2–4^

Most homelessness services aim to help people move towards independent living. Palliative care is not routinely considered unless the individual has a terminal diagnosis or are thought to be at the very end of life.^4^ It can be challenging to know when and how to introduce palliative care as most palliative care services work under the assumption that a person is housed and has a support system around them. When that is not the case, there are added considerations for services to adapt to.

Co-production aims to involve the target audience of research through its entire process.^5,6^ An evolution from traditional consultation Patient and Public Involvement, co-production encourages interactive collaboration.^5^ Co-production recognises the unique perspectives of those with lived experience, and enables them to be team members.^7^ The National Institute for Health and Care Research term this Involvement; ‘where members of the public are actively involved in research projects’.^8^

There has been increased focus on involvement of patients, carers and bereaved individuals across diseases and settings in palliative and end-of-life care research.^9–11^ Studies have identified ‘what works’ in involvement in palliative care research.^12–14^ Recommendations to support researchers to involve people in their research include the recent ‘Patients Changing Things Together’ – a programme to support individuals with terminal illness ‘to lead change that matters to them’.^15^

Further, there is some evidence of co-production with people experiencing homelessness, through community based participatory research.^16^ In Canada, the Lived Experience Advisory Council (LEAC) developed seven principles to underpin inclusion in organisations and initiatives.^17^ Within palliative and end-of-life-care research some projects^18,19^ have involved people with lived experience of homelessness as co-researchers, yet no recommendations for practice exist. General guidance around the involvement of people with lived experience of homelessness has been published by Pathway, a leading UK homelessness charity.^20^ However, this does not focus specifically on palliative and end-of-life-care research. Further, a conceptual framework of co-production mechanisms with vulnerable groups in health service settings has been developed, acknowledging that co-production approaches may require different, in-depth consideration for inclusion health groups.^21,22^

A recent rapid review by our research team identified only one paper outlining the involvement of people with lived experience of homelessness in palliative care research.^23,24^ No papers reported explicit guidance for co-production in this field despite research recommending: ‘There is a need for co-production, combining expertise in palliative care. . .and expertise from homeless and vulnerably housed people’.^21^

This paper presents the TIFFIN recommendations for involving people with lived experience of homelessness in palliative care research. A paper outlining key contextual considerations for co-production in this field^25^ has also been published by this team. which should be considered in combination with the TIFFIN recommendations outlined in this paper.^25^

### Aim

To co-produce recommendations to support the involvement of people with lived experience of homelessness in palliative and end-of-life-care research.

## Methods

### Design

A qualitative study, rooted in a constructive approach producing recommendations based on previous experiences of professionals and people with lived experience of homelessness. Reporting is guided by the Standards for Reporting Qualitative Research.^26^

### Population

Participants were: professionals with experience of involving people with lived experience of homelessness in their work, or people with lived experience of homelessness (Table 1).

**Table 1.** Eligibility criteria for participants.

Participant type	Inclusion criteria
Professional	• Experience of co-producing or involving people with lived experience of homelessness in their work. ‘Involvement’ refers to co-research or co-production, above traditional participation in research. • Working in the field of palliative and end-of-life-care
Person with lived experience of homelessness	• Self-defined previous or current experience of homelessness • Able and willing to articulate views around research involvement in the field of palliative and end-of-life-care

### Recruitment

Professionals were recruited opportunistically; through existing networks and by identifying authors of relevant published literature. Participant group size was determined by the relative infancy of these overlapping fields, resulting in few potential participants to approach. People with lived experience of homelessness were recruited through Groundswell, a peer advocacy organisation and an experienced Peer Coordinator.^27^ When recruiting participants with lived experiences, a description of palliative care in the context of homelessness was discussed with both the peer advocacy organisation and people involved in the project, prior to involvement.

### Data collection

Data were collected between January 2023 and June 2023. Professionals participated in semi-structured interviews via Microsoft Teams discussing their experiences of involving people with lived experience of homelessness in their palliative and end-of-life-care research A semi-structured approach allowed flexibility in discussion, and discussion of issues salient to their experience.^28^

People with lived experience of homelessness were invited to attend one of two online, 90-min focus groups. Discussions surrounded experiences of research involvement, barriers and facilitators to involvement. Participants ‘gave advice’ for researchers planning co-production in this field. Focus groups were organised and facilitated by an experienced Peer Coordinator employed by Groundswell. Discussions were semi-structured using a pre-defined list of prompts. I Interviews and focus groups were audio recorded and transcribed verbatim.

As initial themes and recommendations developed, interview participants were asked to provide feedback via online form. A consultation-based focus group with previous attendees was held to gather further feedback.

### Data analysis

Reflexive thematic analysis was chosen as it ‘emphasises the importance of the researcher’s subjectivity as analytic resource, and their reflexive engagement with theory, data and interpretation’ .^29^.

The six proposed steps for reflexive thematic analysis were worked through by two members of the team (JC and BH).^29^ After data familiarisation, line by line coding produced initial codes. These were constructed into initial themes, which were shared with participants. Interpretative themes were generated through discussion with identified end-users of the research, and the wider research team. Focus group data were analysed inductively, before being combined deductively with the interview themes: any novel or separate findings were highlighted.

### Ethical issues and informed consent

Ethical approval was obtained from University College London (ID: 6202/008). To avoid the small potential for coercion in recruitment, participants had a minimum of 24-h to consider participation. Verbal consent was sought at the beginning of each focus group, by the Peer Coordinator, overseen by an experienced member of the research team. JC is a qualitative researcher within the field of palliative care. Members of the wider research team (BH and CS) have experience in carrying out qualitative research with participants experiencing homelessness. CS is an inclusion health clinician. KF and BH are experienced qualitative researchers within the field of palliative care.

### Patient and Public Involvement

Final themes and recommendations were developed with six people with lived experience of homelessness in a Patient and Public Involvement Consultation. Volunteers were recruited via Groundswell and were reimbursed in line with NIHR Involve’s recommendations, through supermarket vouchers (attendees’ preference).^30^

## Results

Sixteen interviews and two focus groups were carried out with 27 participants (11 people with lived experience homelessness and 16 professionals – Table 2). All people with lived experience were based in the UK.

**Table 2.** Characteristics of study participants.

	Professionals	People with lived experience of homelessness
Gender		
Male	4	4
Female	12	7
Region		
Yorkshire and the Humber	4	–
London	5	–
North West England	2	–
Southwest England	1	–
Birmingham	2	–
Outside of UK	2	–
Organisation type		
University	8	–
Charity	5	–
Hospice	3	–
Job title		
Research fellow	4	–
Registered nurse	2	–
Social worker	2	–
Homelessness liaison worker or coordinator	2	–
Professor	2	–
Research assistant	3	–
Peer coordinator	1	–

Six key themes surrounding best practice for involving people experiencing homelessness in research were identified and associated recommendations for were produced (Table 3).

**Table 3.** Key themes and TIFFIN recommendations.

Theme title	Recommendations
Transparency	• Be transparent regarding the parameters of your project: what aspects cannot be changed (e.g. because they are funder requirements), and where do you have flexibility to make changes through participants’ input? • Consider how you are transparent about the topic of research (i.e. palliative and end-of-life-care). Transparency is key – but needs to be considered in a nuanced and person-centred way, in the context of a longer relationship and provision of on-going support. Homelessness services may not have had much involvement from palliative care teams, so this may be a new area for both staff and service users. • Participants in this study described how they sometimes were not included in projects beyond initial interviews. Offering feedback to co-producers after involvement can demonstrate to them the impact of their involvement. Consider how you can share the results of the study, the impact it may have and the impact their involvement had? If possible, keep them involved through to the end of this process (e.g. involve them in dissemination).
Importance of engagement and rapport	• People experiencing homelessness often have low access to palliative care services. Go to people in the community – do not always expect them to find and come to you. • Try to build genuine trust and rapport, mistrust of services can be common. This can be done by expecting nothing in return before involvement starts. For example, engage in activities together that are unrelated to the research (e.g. going for a walk and doing an activity). • Due to potential previous negative experiences with authority, people experiencing homelessness may lack trust towards academic professionals. Consider using existing links with employed or current co-researchers with lived experience of homelessness to engage. For example, through services with existing links, third-party organisations or through relationships with individuals. Be aware that ‘homelessness’ has different meanings for different people. • Set clear boundaries for involvement. Be clear about what people with lived experience can expect from you, and what you see as part of their involvement. For example, have an informal discussion, or anonymous survey at the start of involvement to find out what people expect so that you can understand any concerns or provide clarity where needed. One key boundary to set can be around confidentiality – where it is upheld and in what circumstances it may need to be broken.
Facilitating equitable involvement via person centred approach	• Prioritise safe, genuine and meaningful involvement. Prioritise the persons’ wellbeing, over the research project. Be aware that co-production can be unpredictable. For example, the timings and order of tasks may need to be flexible and may not follow the typical patterns of traditional academic research. • When carrying out research, try to be flexible and make it easy for people to be involved. Be prepared to throw away your meeting agenda, start early, do not rush and have a plan ‘B’. • Take a person centered approach to transparency in relation to palliative care.
Financial recognition of people’s involvement	• Consider the best way to reimburse people in the context of your project. Ask the people experiencing homelessness what reimbursement type they would prefer? Can accommodate this within your institution? Will this impact people’s benefits?
Involvement and growth: a trauma-informed approach	• Be trauma-informed and aware in your approach to involvement – homelessness and trauma are closely linked. • Ensure there are appropriate supports in place for people with lived experience of homelessness (such as wellbeing and practical skills). For example, consider how you will provide emotional support should a research interaction be triggering for a person with lived experience. • Offer training, coaching or mentoring to support development of research skills. • Encourage reflexive practice at all points throughout involvement: what was learned, what could have been better? This could be done via a conversation, a written piece etc. • Offer support for staff: frequent team debriefs and make efforts to share mental load.
Navigating institutional resistance and attitudes	• Ensuring equitable involvement can mean that traditional approaches need to be challenged. Consider and plan for the amount of time and effort needed to overcome or address institutional challenges for example, payment method, internal advocacy for involvement. • At the end of the project, demonstrate, evidence and share the value of involvement to counter some of the issues faced.

### Transparency

A common perception was that ‘gold standard’ involvement creates powerless relationships between academic and lived experience co-researchers. Participants recognised this hierarchy-free dynamic can be difficult to achieve. It contrasts common perceptions that researchers sit in ‘an ivory tower’, detached from people experiencing homelessness. Academic researchers were concerned that people with lived experience saw them as ‘ironic’ for doing research, from their homes. Although they acknowledged how involvement of lived experience is essential, there was debate around whether the research process is genuinely open for change.

‘Well personally I think we should just cut the bull****. Stop pretending something is power sharing when it isn’t. . . I think what we should be sharing is our resources - our time, our funding, our respect.’ (Research Fellow)

If we cannot truly share ‘power’, it is crucial to be transparent around where input can be impactful and where boundaries are set by funding or research requirements. Participants highlighted that what we do with contributions from people with lived experience is as important as receiving them. It was seen as exploitative to gather contributions without feedback: ‘*They just vanish after taking our notes, and they don’t come back.’ (PWLE). Informing* people with lived experience *post-research can help in demonstrating that their input was respected and valuable.*

*‘Give them the option [of hearing impact of research they were involved in], because then then they will start thinking why is it going to that policymaker? Why is it going in that journal? It teaches them, it can increase confidence, self-esteem.’ (PWLE homelessness)*

*Transparency around the focus of the research is also important to allow potential co-researchers to make an informed decision about their involvement. When operationalised, this may include discussing palliative care in the context of homelessness before involvement begins.*

### Importance of engagement and rapport

Engagement is a fundamental first step and a crucial place for researchers to invest their time and efforts. Sometimes, due to previous negative experiences with people in positions of authority, facilitating engagement can take time, repeated efforts and investment to build trust.

*‘Engagement is over time. It’s not just like this one-off. It’s can you demonstrate trustworthiness? If it’s done well and it’s done sustained a long-term relationship, I think it’s essential.’ (Research Assistant)*

Participants discussed ideas for promoting engagement in meaningful and sustainable ways, for example: building relationships over time and working with community partners (peers). It is important to go into community settings, as opposed to expecting people with lived experience to seek opportunities and approach you: ‘*In my language we say if you don’t go to the seashore, you can’t drink the water’* (person with lived experience). Early engagement activities may be unrelated to the research project, but facilitate the development of trust – a cornerstone for the continued working relationship.

*‘She was doing some gardening with two of them and that’s totally irrelevant to what she’s doing but it’s getting them involved. It’s easing them in gently. . .Even a biscuit doesn’t go a miss. You know, cup of coffee and a biscuit’* (PWLE homelessness)

Established rapport will be helpful for establishing expectations and boundaries for involvement – being clear about where responsibilities start and end for all parties.

### Facilitating equitable involvement via person-centred approach

It is essential to recognise experiences linked with homelessness and to be aware of possible past trauma, without either assuming or dramatising potential needs. Participants emphasised respecting the period of life that people are in, to consider together whether involvement is right for them (e.g. wellbeing and capacity). *‘The people that we’ve been involving certainly in the multiple exclusion homelessness studies they’re often not so far from very traumatic journey points in their life that they’ve escaped. . .So those ongoing relationships will still come up against people who have moved on from addiction, going back to addiction, have moved on from mental health crises, falling back into those crises.’ (Research Associate)*

Often, complex and competing priorities may mean that involvement and ability to engage can fluctuate. It is important to remember that involvement in a project, is just one small part of someone’s life.

*‘I know when I first became homeless I wouldn’t have been able to volunteer anywhere because I just had so many day-to-day issues and I was trying to deal with being made homeless’* (PWLE homelessness).

Flexibility is critical to facilitating equitable, person-centered opportunities for involvement. This was described as challenging ‘normal’ academic practice (i.e. how research without involvement might typically be conducted), to introduce flexibility around aspects of involvement (such as allowing extra time, and considering the physical and literacy-based accessibility of your project).

*‘If they’re [LE co-researcher] having a bad day, and they’re not in the mood to talk, give them another day. You have to take into consideration how they’re feeling’* (person with lived experience).

### Financial recognition of people’s involvement

There was consensus regarding the provision of reimbursement, and ensuring projects are ‘*generously costed*’ (*Professor)* to enable this. Some participants saw cash payments as the ideal, and *‘a universal token, that gives that person the freedom to spend it on anything’ (Professor).* .

*‘Always cash. Cash in hand. . .We’re asking for their time and expertise. It’s same as anybody else. That gatekeeping and gift cards. . . it’s none of your business what people use their money for.’ (Research Assistant)*.

Professionals described how rigidity in organisations systems didn’t always allow for cash payments. Instead, vouchers were often used. To counter this, some organisations liaised with external organisations to organise payments beyond vouchers, where this was the person with lived experience’s preference.

*‘So [person in external organisation] claimed the money and the money went to her and she’s administered it. Obviously she has to send us a breakdown of the cost and she keeps her own accounts and then sends that to us as part of our submission and our reporting.’ (Research Fellow)*

People with lived experience agreed that financial recognition was always valued: *‘They’re rewarding you, valuing your time in a way, so you don’t feel exploited’ (PWLE homelessness).* One gave an example of reimbursements impact:

‘*I got like a hundred quid, and it was the first time in [pause] . . ., I think I’ve been housed now for about five or six years, but it was the first time in that period that I actually had a fridge and cupboard full of food and I wasn’t just eating crap. So it helps dramatically.’* (PWLE homelessness)

However, it is critical that involvement doesn’t impact people’s financial security through impacting on receipt of benefits.

*‘There’s just the whole bundle of issues around Universal Credit. Some people don’t want to get paid because it makes it really hard to get your benefits. £40 dropping into your account from unknown sources is the sort of thing that gets the Department for Work and Pensions a bit wound up, unfortunately.’ (Research Fellow)*

### Involvement and growth – A trauma-informed approach

Both researchers and people with lived experience expressed the necessity of a trauma-informed approach to involvement. Trauma experienced by many people with lived experience of homelessness is intertwined with experiences of death and bereavement, thus involvement in palliative care research may resurface emotions and trauma.

*‘Sometimes [involvement] makes you in pieces because you are going back to that time- Your mind is saying, let’s move on, but sometimes when you put shoes in that time it breaks you. So aftercare is very, very important’ (PWLE homelessness)*

People with lived experience described the importance of having secure plans in place to deal with potential distress and avoiding re-traumatisation. This included ongoing coaching, training prior to involvement and frequent check-ins.

*‘You should be offering people coaching before they even go into doing the research. You can’t just throw people into becoming researchers without some kind of training. . .Part of being trauma-informed is offering coaching’ (PWLE homelessness)*

Participants also discussed the need to allow people with lived experience to guide the direction and intensity of involvement. Further, recognising that what is traumatic, is defined by the individual: researchers may be shocked by an experience that to the person with lived experience, is normalised.

‘*I think one thing to bear in mind, you could hear something, and it may really impact you. And you’re like, surely this person doesn’t want to continue this conversation after the things they’ve just said. Whereas for that person it’s something that they’ve dealt with for days, weeks, and months. So, to you it’s like whoa, this is really impacting. But you know they’ve possibly already dealt with that.’ (PWLE homelessness)*

### Navigating institutional resistance and attitudes

Professionals discussed the structural, institutional challenges related to involvement-led research.

*‘If you’re truly involving people with lived experience in a way that’s really meaningful to them, then the normal rules that academia imposes don’t count.’ (Research Assistant)*

Professionals described witnessing stigma or assumptions around homelessness, including being ‘*too dangerous or too vulnerable’* to be involved in palliative and end-of-life-care research *(Research Fellow*). Interviewees suggested structural challenges may exist, or be worsened, as a result of such stigma. For example, ingrained views that people experiencing homelessness may make choices others may consider unwise if they are reimbursed with cash. This could influence institutional decisions about methods of payment. Further, even without stigma, University systems often don’t allow for cash payments to be made, introducing restrictiveness into the research process.

*‘All the systems and structures that we have that are really prohibitive around paying people, employing people, consulting with people. . .You really have to rethink all of those and that’s a challenge. . .it’s a challenge often to the team, but it’s also a challenge to the academic structures that are around that team. So, it’s a lot of extra work and hassle with people who are used to doing things a certain way.’ (Research Fellow).*

Although issues may be specific to an institution, building an evidence base for why coproduction and involvement is essential to counter this. This recommendation aims to highlight these issues so researchers can be ready to challenge them to enable involvement of people with lived experience of homelessness. Recognising stigma and assumptions can be key to challenging them, without stifling discussions around nuances and safety.

## Discussion

### Main findings/results of the study

This study explores the experiences of people with lived experience of homelessness and associated professionals to co-produce recommendations around involvement in palliative and end-of-life-care research. The TIFFIN recommendations could support safe and meaningful involvement and should be considered within the institutional context of the research.

Six themes were identified: Transparency; Importance of Engagement and Rapport; Facilitating equitable involvement via person-centred approach; Financial recognition of people’s involvement; Involvement and growth – a trauma-informed approach and Navigating institutional resistance and attitudes. These were developed using insights from the field of palliative and end-of-life-care, though could be applicable to other forms of health research.

### What this study adds?

Previous research has either looked at co-production of palliative care research, or involvement of people with lived experience of homelessness in wider research; to the best of the authors knowledge, there is no research providing guidance for how to consider both together.

Aspects of the TIFFIN recommendations echo key principles of involvement in healthcare research more generally, such as ‘Acknowledge, reward and value everyone involved’.^31^ The context of homelessness adds complexities, for example consideration of whether reimbursement might impact people’s benefit payments. Furthermore, Shippee et al.^32^ reported four components of patient and service user engagement that reflect broadly on those proposed in the TIFFIN recommendations. Therefore, the TIFFIN recommendations build upon existing.^32^ knowledge surrounding general healthcare co-production practices, while considering the need for a nuanced, trauma-informed approach.

Some of the TIFFIN recommendations echo sentiments within reviews of patient and carer involvement in palliative care research in the general population,^12^ such as: ‘acknowledge difficulties; however, do not make assumptions’; ‘take time to create a safe space and to work together collaboratively, acknowledging boundary issues’ and ‘Flexible approaches to involvement’ and ‘Building and maintaining relationships’.^13^ Palliative care support for people experiencing homelessness is still developing. Many of the TIFFIN recommendations will be applicable to people who have not experienced homelessness, but importantly, they include considerations that support person centred and trauma informed approaches to involvement. Whilst these recommendations have been developed in the context of homelessness, it would be beneficial to explore whether they may need refinement for other inclusion health populations.

Linked to this, it is crucial to recognise the need for comprehensive support throughout projects. For example, ensuring co-researchers are supported to explore what palliative care means in the context of inclusion health and homelessness. Considering the unique situation of each research team and project is important. To facilitate safe and meaningful involvement, drawing on existing partnerships or building new relationships to tap into resources that already exist is useful, for example organisations well suited to supporting people with lived experience of homelessness. It is important for researchers to recognise where their own skillsets begin and end in order to promote safe involvement for everyone.

### Strengths and weaknesses/limitations of the study

#### Strengths

Currently there is little involvement of people with lived experience of homelessness in palliative and end of life care research. The TIFFIN recommendations offer practical steps to begin to address this important gap. This study was supported by people with lived experience of homelessness throughout. Iterative input was gathered from 15 people with lived experience. This process improved and validated the recommendations.

#### Limitations

Due to small number of academic researchers actively co-producing research in this field, the pool of professionals to recruit from was small. However, participants represented a range of career stages and experiences. People with lived experience were approached by Groundswell’s staff. This approach builds upon existing relationships and support structures but limits our insights into any possible non – responder bias.

A further obstacle relates to identification of who, among a population of people who tend to have very poor health and limited engagement with health care services, could benefit from a palliative approach. Previous research^33^ suggests concern about a person’s deterioration in health could act as a trigger for considering palliative care support, rather than the receipt of a terminal diagnosis. To accommodate this uncertainty, rather than using diagnoses or other fixed criteria to indicate when someone’s lived experience may be relevant for research in this field, we would advocate for self-identification of relevant lived experience.

Discussing a palliative approach to care, and emphasising its supportive, multidisciplinary nature with people with lived experience of homelessness and staff can counter some of the misconceptions about palliative care and facilitate involvement in palliative care research.

## Conclusion

As a population with high levels of premature morbidity yet low access to palliative care, more work needs to be done to advocate for the involvement of people with lived experience of homelessness in palliative and end-of-life-care research. The TIFFIN recommendations can act as a guide to supporting the involvement of this group in palliative and end-of-life-care research in order to reduce the inequity that currently exists in end-of-life experiences.
